# Vulvar Crohn’s disease in an adolescent diagnosed after unsuccessful surgical treatment

**DOI:** 10.1186/s12905-021-01449-4

**Published:** 2021-08-28

**Authors:** Suhra Kim, Young Bin Won, Seok Kyo Seo, SiHyun Cho, Young Sik Choi, Byung Seok Lee, Bo Hyon Yun

**Affiliations:** 1 Department of Obstetrics and Gynecology, Severance Hospital, Yonsei University College of Medicine, 50 Yonsei-ro, Seodaemun-gu, Seoul, 03722 Republic of Korea; 2 Institute of Women’s Life Science, Yonsei University College of Medicine, Seoul, Korea; 3 Department of Obstetrics and Gynecology, Gangnam Severance Hospital, Yonsei University College of Medicine, Seoul, Republic of Korea

**Keywords:** Metastatic Crohn’s disease, Vulvar Crohn’s disease, Vulvar inflammation, Vulvar abscess

## Abstract

**Background:**

This case report presents a case of Vulvar Crohn’s disease (VCD) in an adolescent, that is an uncommon manifestation of Crohn’s disease (CD) without gastrointestinal symptoms. Before treating CD itself with proper medication, vulvar abscess continued to recur without improvement.

**Case presentation:**

We report the case of an 18-year-old woman with VCD. After treatment with azathioprine 50 mg daily and mesalazine 1 g three times daily, vulvar lesions resolved after 6 weeks. We collected electronic medical data on patient characteristics, and evaluated findings of physical examinations, pelvic MRI, and biopsy specimen obtained from gastroduodenoscopy/colonoscopy.

**Conclusions:**

VCD is a rare manifestation of CD that may be misdiagnosed in the absence of gastrointestinal symptoms leading to delayed treatment. If a patient has an unexplained vulvar inflammatory lesion and with repeated failed surgical treatment, gynecologists should consider the possibility of a VCD.

## Background

Crohn’s disease (CD) commonly manifests as a chronic inflammatory bowel disease presenting with gastrointestinal symptoms, such as diarrhea, dyspepsia, and perianal complication [1], but also rarely manifests extra-intestinally on the vulva without evidence of intestinal CD [2]. The two forms of vulvar Crohn’s disease (VCD) presentation include lesions that are contiguous with the gastrointestinal tract, such as fistulas and fissures; the other form comprises of noncontiguous vulvar lesions, referred to as metastatic CD [3]. Unfortunately, due to the rarity of VCD, patients may not be diagnosed accurately or diagnosis is initially delayed, and the patient may unintentionally receive unsuccessful localized surgical treatment [4, 5]. Even incidence of VCD is still unrevealed because of its rarity.

We present a case of VCD, an uncommon manifestation of CD, in an adolescent who did not have any gastrointestinal symptoms. Delayed diagnosis led to delayed treatment and unnecessary surgical interventions. Relevant literatures were reviewed from search data in PubMed using the key words ‘‘vulvar Crohn’s disease, metastatic Crohn’s disease, vulvar inflammation.” and a brief summary of cases are shown in Table 1  [2–4, 6–12].

**Table 1:** Summary of VCD cases in clinical, histological, Imaging findings and management

Clinical presentation	Asymmetric/symmetric vulvar swelling Vulvar pain Aphthoid or linear “knife-like” vulvar ulcer (often extends to groin) Hypertrophic exophytic vulvar lesion/ Scarring, Plaque, Pedunculated tags of tissue Vulvar abscess with sinus tract Vesicles, Papules/nodules at vulva Vaginal discharge Vulvar itching Perianal skin tags/fissure
Histological finding of vulvar lesion	Noncaseating granuloma Mixed inflammatory cell infiltrates Fibrosis Dilatation of lymphatics and capillary vessels Hyperkeratosis Dermal thickening Vascular ectasia
*Imaging study*	
Pelvis MRI	Rectovaginal/Perianal fistula Perineum abscess Perineum marked edema Perineum thickening Small volume lymphadenopathy with evidence of fissuring in the perianal region.
Abdomen-Pelvis CT	Vulvar abscess and surrounding inflammation
Perineal sonography	Diffuse hypoechogenicity Increased dermal thickness Diffusely elevated color doppler signal by local inflammation
Management	Corticosteroid Immunosupressant

MRI, magnetic resonance imagning; CT, computed tomography

We collected electronic medical data on patient characteristics, and evaluated findings of physical examinations, pelvic MRI, and biopsy specimen obtained from gastroduodenoscopy/colonoscopy.

## Case presentation

**Fig. 1:**
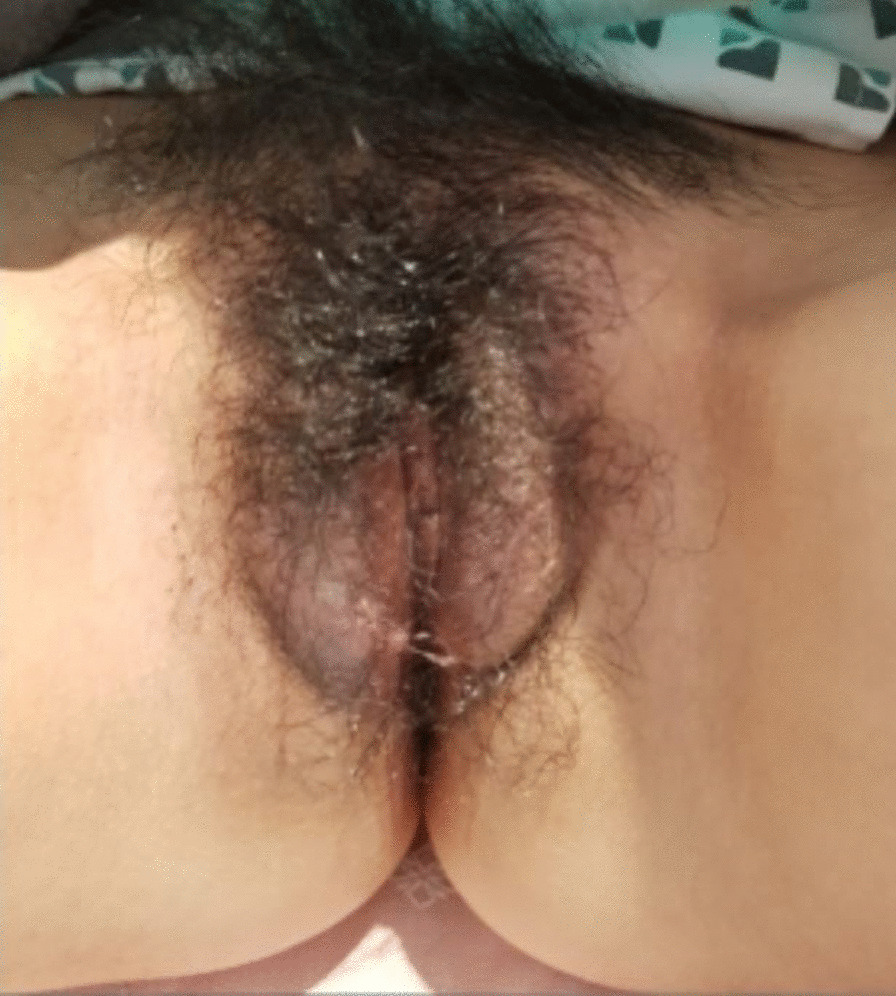
Bilateral vulvar swelling with abscess. Gross picture at the time of first visit: Bilateral swollen vulva with abscess-like discharge was noticed, although the patient has been undergone the I&D 3 times

**Fig. 2:**
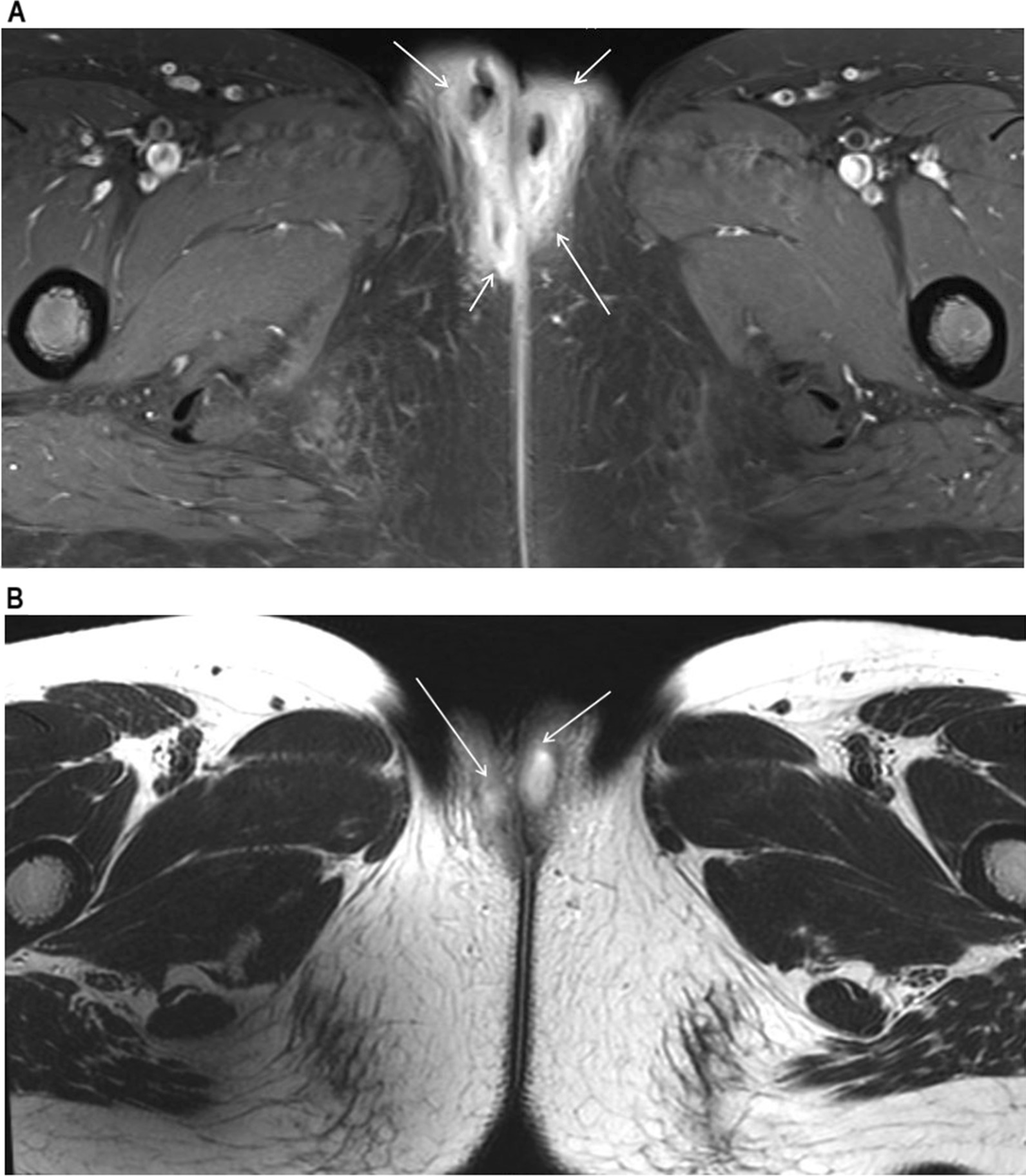
MRI image showing vulvar abscess. **A** Axial view of pelvic MRI prior to I&D at previous hospital with soft tissue infiltration and enhancement in both labia major and perineum (arrow: soft tissue inflammation and swelling with abscess). **B** Follow-up MRI taken 6 weeks postoperatively at our institution (arrow: abscess)

An 18-year-old woman without any significant history of previous illnesses visited an outpatient clinic with a chief complaint of recurrent vulvar abscess for 5 months. On inspection, the vulva was diffusely swelling, tender, red and warm (Fig. 1). The patient had no other complaints apart from perineal pain and difficulty in sitting for a long time. She had previously visited another hospital with the same complaint, and on 3 occasions received incision and drainage (I&D) of the abscess. She visited a secondary hospital, where abdominal and pelvic computed tomography (APCT) showed a labia major abscess and laboratory results showed no abnormalities. She received antibiotic treatment, comprising of cephalosporin and metronidazole for 2 weeks, but her symptoms did not improve. A magnetic resonance imaging (MRI) of the pelvis was performed which revealed a 9 × 7 mm sized abscess in the right labia major (Fig. 2A). Under the impression of persistent vulvar abscess with positive culture results implicating *Citrobacter freundii, Corynebacterium striatum*, and *Eschericia coli*, I&D with local excision of inflamed tissues was performed under general anesthesia. The pathology results showed acute and chronic inflammation with granulation tissue formation, without indication of caseation status. Intravenous antibiotics were administered for 2 weeks and the vulvar inflammation subsided. However, inflammation and painful swelling of the contralateral labia major arose 2 weeks postoperatively.

She was referred to our institution, a tertiary care hospital. At the pediatric and adolescent gynecology clinic, a follow-up MRI was performed because the features of the abscess were not typical of a gynecological abscess. A deep pelvic abscess, at a higher level than Bartholin abscess, was suspected. The MRI results showed diffuse inflammation of the perineum around the posterior vaginal wall with abscess formation along both vestibular glands as well as both labia major and minor. There were also features suggestive of a rectovestibular fistula at the 12–1 ‘o’clock position (Fig. 2B). The patient was then clinically evaluated for Crohn’s disease because of the above MRI findings. Her laboratory tests, including inflammatory markers, were normal.

**Fig. 3:**
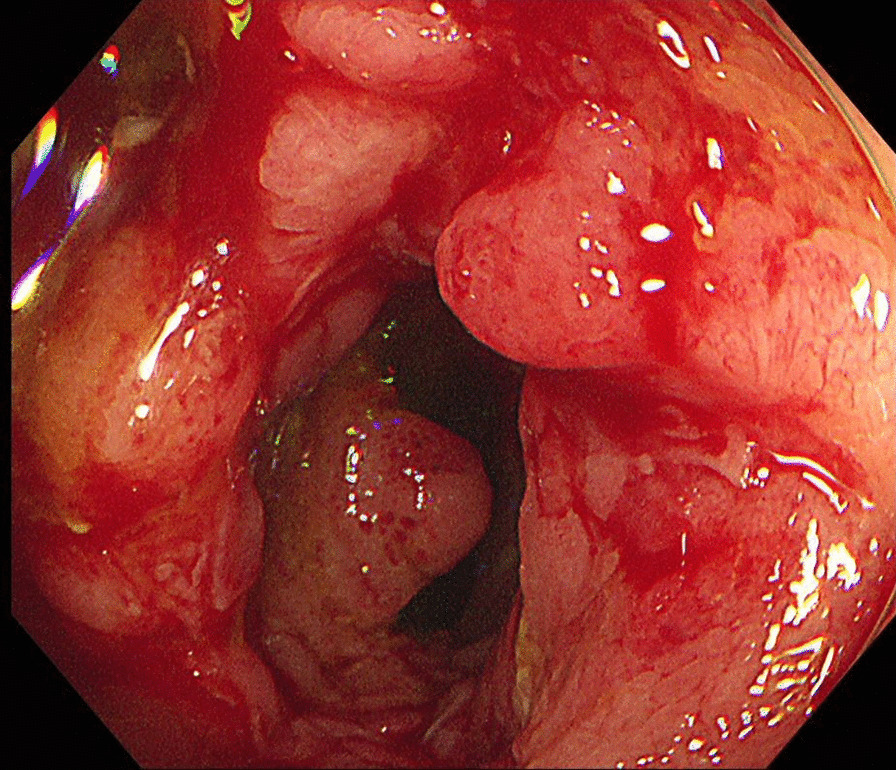
Crohn’s ulcers on colonoscopy. Multiple ulcers were seen on terminal ileum mucosa

The patient was then referred to the pediatric gastroenterology department where she underwent further blood tests, gastroduodenoscopy, and colonoscopy to rule out other differential diagnoses, such as sarcoidosis, pyoderma gangrenosum, hiradenitis suppurativa, cellulitis, tuberculosis, and contact dermatitis. Stool calprotectin levels was elevated to > 300 µg/g, which suggested a diagnosis of inflammatory bowel disease. A more detailed history revealed that the patient had no history of diarrhea or hematochezia. On gastroduodenoscopy, gastric mucosa was noted to be erythematous which was suggestive of reflux esophagitis and chronic superficial gastritis. Colonoscopy revealed multiple ulcers on the mucosa of the terminal ileum and the rectum (Fig. 3). Pathological evaluation of tissue specimen retrieved showed mild chronic superficial gastritis and ulceration with ill-defined noncaseating granulomatous lesions in the mucosa of the terminal ileum, which were consistent with a diagnosis of Crohn’s disease or tuberculosis. Further AFB staining and Tb-PCR performed on biopsy samples taken from the terminal ileum were negative, which ruled out tuberculosis. The rectal biopsy specimen was within normal limits. The simple endoscopic score for Crohn’s disease was 11 [13]. The result of the stool culture was positive for *Clostridium difficile* and the patient received oral metronidazole therapy.

**Fig. 4:**
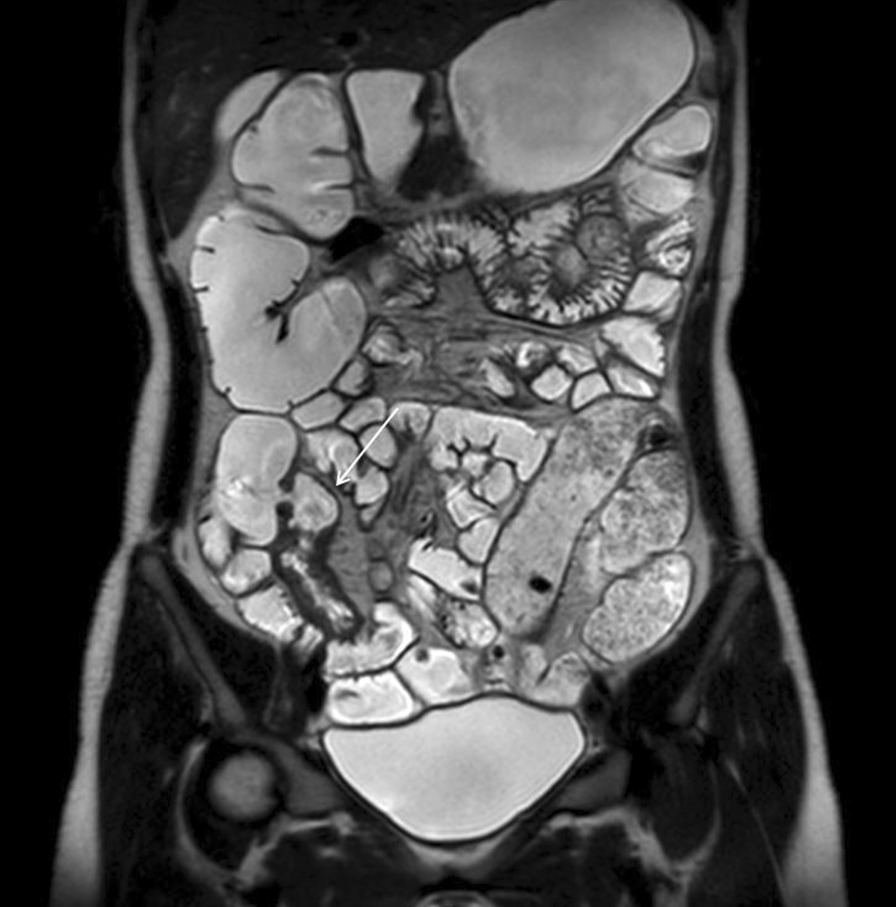
MR enterography. Coronal view of the patient’s MRI enterography with segmental and uneven wall thickening of ulcerative lesions in distal to terminal ileum (arrow)

MRI enterography after oral contrast ingestion showed segmental and uneven wall thickening with ulcerative lesions from the distal to the terminal ileum and distal rectum with increased mucosal enhancement and diffusion restriction (Fig. 4). Diffuse bilateral perineal soft tissue infiltration and increased enhancement with features suggestive of a rectovaginal (11 ‘o’clock) and a vaginoperineal (bilateral anterior) fistula were also observed. The abscess in both vestibular glands and both labia were smaller in size compared to those in the pelvic MRI performed the previous month at the gynecology department. The Pediatric Crohn’s Disease Activity Index (PCDAI) was 12.5, which indicated clinical response (≤ 12.5), but not inactive disease (< 10) [14]. The modified PCDAI score was 2.5, indicating remission (< 7.5) [15].

She was placed on elemental diet (2000 kcal/day) four times daily for 12 weeks, azathioprine 50 mg daily, and mesalazine 1 g three times daily. Azathioprine was increased to 75 mg daily after three weeks. The elemental diet was stopped after the prescribed 12 weeks, and she was maintained on 75 mg daily of azathioprine. The vulvar lesion completely resolved and her white blood cell (WBC) count was 3470/µL during her follow-up visit after 6 weeks of treatment. Inflammatory markers were normal, and the PCDAI was 10. Oral Azathioprine was increased to 100 mg daily, with continuous WBC monitoring on follow-up.

**Fig. 5:**
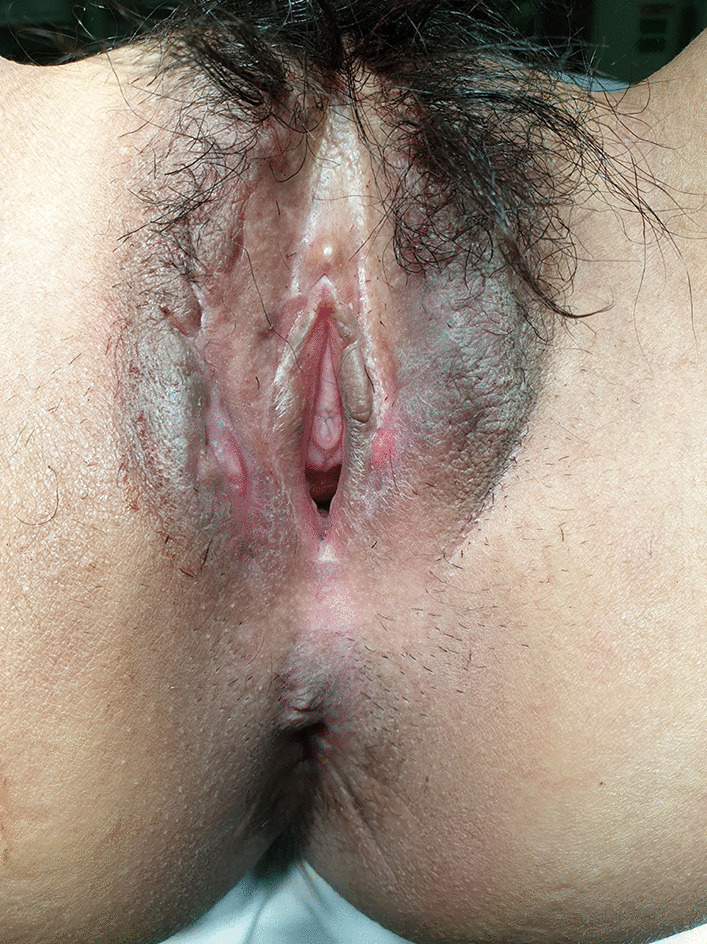
Improvement of vulvar lesion after medical treatment of Crohn’s disease. Vulvar swelling is remained slightly, but abscess has been almost resolved after starting azathioprine treatment

**Fig. 6:**
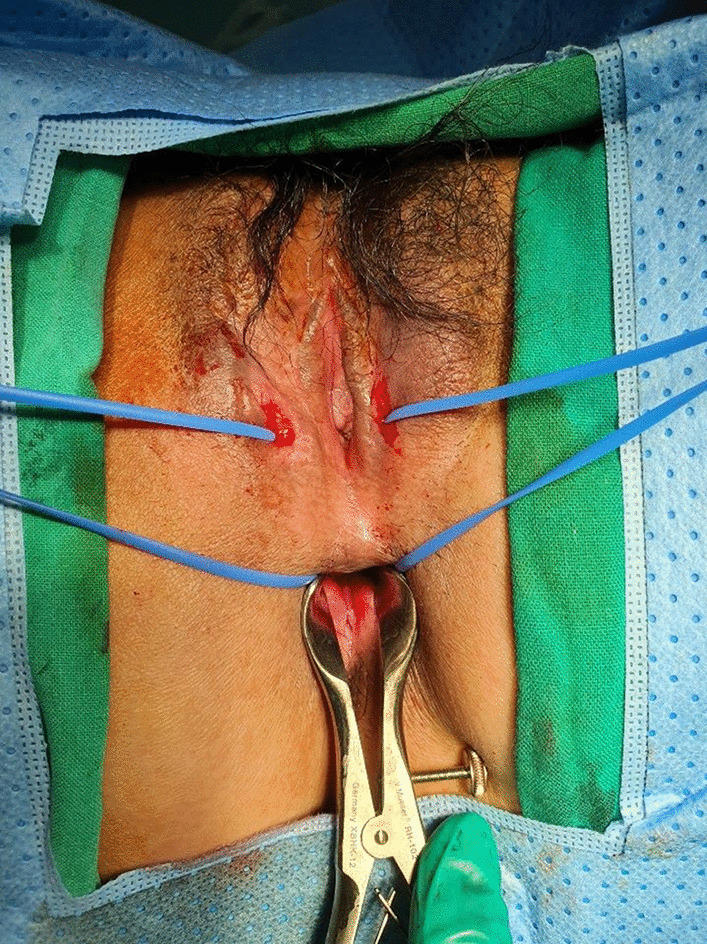
Changes of vulvar lesion after recto-vaginal fistula treatment. Seton procedure was performed to treat rectovaginal fistula, which caused sustained inflammation of vulvar area

The patient revisited the emergency room with vulvar pain after taking medication for Crohn’s disease for 10 weeks. On examination, there was right labia major swelling, tenderness, and pus discharge (Fig. 5). She underwent a Seton procedure for rectovaginal fistula (Fig. 6). While her symptoms initially improved, a recurrence required second operation. About 4 months after re-operation, her symptoms finally resolved.

## Discussion and conclusion

VCD is a rare manifestation of metastatic CD in which inflammatory granulomatous lesions are separated from the gastrointestinal tract [4]. It manifests frequently as vulvar edema, ulcers, and fissures. Vulvar symptoms may be the first and only symptom of such patients [6]. Unfortunately, VCD is an uncommon presentation of Crohn’s disease, and not all patients present with active gastrointestinal disease, and this leads to delays in making the right diagnosis [6, 16]. Less than 200 cases of VCD have been published so far [7–9, 17]. These cases of VCD are usually accompanied by gastrointestinal fistulas, but most do not undergo gastrointestinal evaluation when they do not present with recognizable gastrointestinal symptoms [18–20]. They are usually diagnosed at adulthood, with a mean age at diagnosis of 34 years [4], and only a few cases have been diagnosed in children [10, 21, 22].

Many differential diagnoses must be considered, including Behcet’s disease, cellulitis, pyogenic infections, hidradenitis suppurativa, sarcoidosis, tuberculosis, foreign body reactions, contact dermatitis, acquired lymphangiectasia, and sexual abuse before a diagnosis of VCD can be made [10]. Results of pathological evaluation of gastrointestinal biopsy specimens are vital in making a diagnosis of VCD. Not every patient with vulvar symptoms should undergo gastroduodenoscopy or colonoscopy as a routine work-up; however, it should be considered in patients with atypical disease features. In VCD patients, pelvis MRI or anorectal endoscopic ultrasound may be helpful to identify rectovaginal fistula complex. In our case, we could see rectovaginal fistula on pelvic MRI and abscess was located higher, deeper than usual abscess from gynecologic origin. Moreover, in recurrent vulvar abscess with rectovaginal fistula combined to VCD, vulvar malignancy should be excluded by biopsy as well. In some VCD cases, vulvar cancer rising from recurrent abscess with fistula was reported. In our case, histologic report from vulvar abscess revealed acute and chronic inflammation, which helped to exclude malignancy eventually. Although a diagnosis of VCD is made, if recurrent vulvar abscess develops still, the patient should be monitored and informed about the possibility of malignancy at her vulvar lesion [20, 23, 24].

In current case, the patient had no gastrointestinal symptoms, but vulvar symptoms only. She had few event of loose stool or diarrhea, irritable bowel symptom. The strength of our report includes that focal vulvar abscess which does not respond to the conventional treatment, although patient has none of bowel symptoms, Crohn’s disease must be included for the differential diagnosis. The atypical manifestation of VCD without gastrointestinal symptom is unique for our case report.

The treatment of VCD focuses on the standard treatment of Crohn’s disease, such as corticosteroids, metronidazole, and azathioprine, which has been observed to result in varying degrees of success in treating vulvar lesions [2]. The diagnosis of VCD may be delayed and the disease might not be properly treated; therefore, tumor necrosis factor-ɑ inhibitors, such as infliximab, have been recommended for the treatment of refractory vulvar symptoms [6, 16]. Early diagnosis and treatment are key, as delayed treatment may lead to permanent vulvar distortions and decreased quality of life. Surgical treatment may be considered as a last resort if the vulvar lesions do not respond to medical treatment; however, this frequently results in localized recurrence [5, 9].

The management of VCD is challenging as it is a rare disease with nonspecific symptoms, that requires close cooperation from gynecologists, dermatologists, and gastroenterologists alike [3]. Our experience reported in this case report should guide gynecologists to consider and suspect a vulvar presentation of CD in cases of unexplained vulvar inflammatory lesions that are unresponsive to antibiotics or surgical treatment.

## Data Availability

Data sharing is not applicable to this article as no datasets have been generated. Moreover, according to the nature of the case report, written informed consent is not including further data sharing to others.

## Abbreviations

APCT: Abdominal and pelvic computed tomography
CD: Crohn’s disease
I&D: Incision and drainage
MRI: Magnetic resonance imaging
PCDAI: Pediatric Crohn’s disease activity index
VCD: Vulvar Crohn’s disease
WBC: White blood cell
